# Coexistence of the *bla*_NDM-1_-carrying plasmid pWLK-NDM and the *bla*_KPC-2_-carrying plasmid pWLK-KPC in a *Raoultella ornithinolytica* isolate

**DOI:** 10.1038/s41598-020-59341-4

**Published:** 2020-02-11

**Authors:** Bingjun Dang, Haoyang Zhang, Ziwei Li, Shuanglong Ma, Zicheng Xu

**Affiliations:** 1 College of Tobacco Science, Henan Agricultural University, Zhengzhou, 450002 China; 2 College of Resources and Environmental Sciences, Henan Agricultural University, Zhengzhou, 450002 China

**Keywords:** Microbiology, Environmental microbiology, Water microbiology

## Abstract

To date, *bla*_NDM_ and *bla*_KPC_ genes have been found predominantly in clinical settings around the world. In contrast, bacteria harbouring these two genes from natural environments are relatively less well studied compared to those found in clinical settings. In this study, a carbapenem-resistant *Raoultella ornithinolytica* strain, WLK218, was isolated from urban river sediment in Zhengzhou City, Henan Province, China. This isolate was subjected to PCR and antimicrobial susceptibility testing. PCR results showed that this isolate was positive for both the *bla*_NDM-1_ and *bla*_KPC-2_ genes. The antimicrobial susceptibility testing results showed that this isolate exhibited resistance or intermediate resistance to all the antibiotics tested except for streptomycin (susceptible) and cefepime (susceptible-dose dependent). The complete genome sequence of the WLK218 isolate was then determined by using a combination of the PacBio and Illumina sequencing technologies. The *de novo* assembly of the genome generated one chromosome and six plasmids. Among the six plasmids, the *bla*_NDM-1_ gene was carried on the IncX3 plasmid pWLK-NDM, while the *bla*_KPC-2_ gene was located on the untypeable plasmid pWLK-KPC. This is the first report of an environmental *Raoultella ornithinolytica* isolate co-harbouring the *bla*_NDM-1_ and *bla*_KPC-2_ genes.

## Introduction

The increasing prevalence of multidrug-resistant *Enterobacteriaceae* pathogens has rendered life-saving antibiotics less effective, which has required clinicians to increasingly use last-resort antibiotics, such as carbapenems and polymyxins. However, the emergence and spread of antibiotic resistance genes (ARGs) conferring resistance to last-resort antibiotics have been observed across the globe in recent years, which has posed a challenging threat to public health^1–6^.

As one of the ARGs conferring resistance to last-resort antibiotics, the *bla*_NDM-1_ gene has gained worldwide attention because the carbapenemase encoded by this gene can hydrolyse nearly all classes of β-lactams (including carbapenems), with the exception of monobactams^7^. The *bla*_NDM-1_ gene was first discovered in *Klebsiella pneumoniae* and *Escherichia coli* clinical isolates in India in 2008^8^. Just after the initial detection in clinical settings, *bla*_NDM-1_-carrying isolates were soon found to be widely distributed in surface waters in the environment of New Delhi^9^. Since then, *bla*_NDM_ genes have been extensively studied in clinical settings. *Klebsiella pneumoniae* carbapenemases (KPCs) are another type of carbapenemases which have also gained worldwide attention. The first member of KPC family, namely KPC-1, was identified in a *Klebsiella pneumoniae* clinical isolate in the USA in 1996^10^. Afterwards, KPC-producing bacteria have spread rapidly internationally, especially in China^11^. The situation of KPC in China is very serious. It is reported that China is one of the KPC endemic countries in the world^11,12^.

In China, environmentally isolated *bla*_NDM_-carrying and *bla*_KPC_ carrying strains have been found in different environmental samples, such as poultry production environments, hospital sewage systems and wastewater, and river sedimens^13–17^. However, studies in environmental settings are still relatively lacking at present. In the present study, we report the isolation of a *Raoultella ornithinolytica* strain co-harbouring the *bla*_NDM-1_ and *bla*_KPC-2_ genes from urban river sediment. To the best of our knowledge, this is the first report of an environmental *Raoultella ornithinolytica* isolate co-harbouring the *bla*_NDM-1_ and *bla*_KPC-2_ genes. Furthermore, we determined the complete genome sequence of this strain through whole genome sequencing. Comprehensive sequence analysis was conducted to gain insight into the genetic structures and organizations of the plasmids that harboured the *bla*_NDM-1_ and *bla*_KPC-2_ genes.

## Materials and Methods

### Study sites and sample collection

Sediment samples were collected from the urban river systems of Zhengzhou City, Henan Province, China. The sampling site (WLK) was located downstream from the confluence of the urban river and the WuLongKou WWTP discharges. After sample collection, the sample was stored at −20 °C prior to further analysis.

### Bacterial strains

*Raoultella ornithinolytica* strain WLK218 was recovered from the WLK sample. Briefly, Ten-fold serial dilutions of the sediment sample were prepared, and 100 µl of each dilution was plated on LB agar plates supplemented with 5 mg L^−1^ meropenem. The plates were incubated at 30 °C for 2 days. After incubation, morphologically distinct single colonies were picked and then streaked continuously in the same selective medium to purify the meropenem-resistant bacteria. Bacterial species identification was conducted by using 16 S rRNA gene sequencing. The carbapenem resistance genes were detected by PCR, followed by sequencing. All the sequencing results were confirmed by BLAST analysis. Detailed information about the PCR primers used in this study is summarized in Table S1 of the Supplementary Materials.

### Antimicrobial susceptibility testing of the *Raoultella ornithinolytica* strain WLK218

The antimicrobial susceptibilities of *Raoultella ornithinolytica* strain WLK218 were initially tested by using the Kirby-Bauer disk diffusion method on Mueller-Hinton agar plates with 10 antibiotics (ampicillin, gentamicin, streptomycin, tetracycline, imipenem, meropenem, ertapenem, erythromycin, kanamycin, and trimethoprim/sulfamethoxazole) according to the criteria of the Clinical and Laboratory Standards Institute (CLSI). We further determined the minimum inhibitory concentrations (MICs) of amikacin, ampicillin, ampicillin/sulbactam, aztreonam, cefazolin, cefepime, cefotetan, ceftazidime, ceftriaxone, cefuroxime, ciprofloxacin, gentamicin, imipenem, levofloxacin, meropenem, nitrofurantoin, piperacillin, piperacillin/tazobactam, tobramycin, and trimethoprim/sulfamethoxazole by using a VITEK 2 automated system with AST GN09 cards (bioMérieux, France). The results generated by the VITEK 2 system were interpreted according to the clinical breakpoints defined by the CLSI (M100-S29)^18^. *Escherichia coli* ATCC25922 was used as the quality control strain for antimicrobial susceptibility testing.

### Whole genome sequencing, assembly and data analysis

For whole genome sequencing, total genomic DNA was prepared from the *bla*_NDM-1_-positive isolate with the DNeasy Blood and Tissue Kit (Qiagen, Germany) following the manufacturer’s instructions for Gram-negative bacteria. The concentration and purity of the extracted DNA were determined by a Qubit fluorometer and a NanoDrop, respectively. Then, the qualified DNA was used for whole genome sequencing. Whole genome sequencing was carried out with a combination of the PacBio Sequel (Pacific Biosciences, Menlo Park, CA, USA) and HiSeq X Ten (Illumina, San Diego, CA, USA) sequencing platforms at the Beijing Genomics Institute (BGI, China). Subsequently, all the raw reads were quality-trimmed, and the obtained high-quality PacBio reads were subjected to self-correction and Illumina-based correction. The corrected PacBio reads were *de novo* assembled with a combination of Celera Assembler (version 8.3) and Falcon (version v0.3.0). After being quality-checked with Quiver and making single-base corrections in SOAPsnp/SOAPindel and GATK (version v1.6-13), the assemblies were circularized to generate circular chromosomes and plasmids with no existing gaps.

Gene prediction was carried out using Glimmer (version 3.02) followed by manual inspection and refinement. The annotations of the predicted genes were performed by BLASTP. Plasmid replicons, ARGs and mobile elements were identified by using online databases, including PlasmidFinder (https://cge.cbs.dtu.dk/services/PlasmidFinder), BacWGSTdb (http://bacdb.org/BacWGSTdb)^19^, ISfinder (http://www-is.biotoul.fr) and the Tn Number Registry (http://www.ucl.ac.uk/eastman/research/departments/microbial-diseases/tn).

### Nucleotide sequence accession numbers

The complete sequences of the *Raoultella ornithinolytica* strain WLK218 chromosome, pWLK-NDM and pWLK-KPC were submitted to GenBank under accession numbers CP038281, CP038280 and CP038279, respectively.

## Results

### Characterization of the *Raoultella ornithinolytica* strain WLK218

Through PCR assays, strain WLK218 was found to be positive for both the *bla*_NDM-1_ and *bla*_KPC-2_ genes. The antimicrobial susceptibility testing results showed that the *Raoultella ornithinolytica* strain WLK218 exhibited resistance or intermediate resistance to meropenem (R, ≥ 16 mg/L), imipenem (R, ≥ 16 mg/L), amikacin (R, ≥ 64 mg/L), ampicillin (R, ≥ 32 mg/L), ampicillin/sulbactam (R, ≥ 32/16 mg/L), aztreonam (R, ≥ 64 mg/L), cefazolin (R, ≥ 64 mg/L), cefotetan (R, ≥ 64 mg/L), ceftazidime (R, ≥ 64 mg/L), ceftriaxone (R, ≥ 64 mg/L), cefuroxime (R, ≥ 64 mg/L), ciprofloxacin (R, 2 mg/L), gentamicin (R, ≥ 16 mg/L), piperacillin (R, ≥ 128 mg/L), piperacillin/tazobactam (R, ≥ 128/4 mg/L), tobramycin (R, ≥ 16 mg/L), trimethoprim/sulfamethoxazole (R, ≥ 320(16/304) mg/L), levofloxacin (I, 1 mg/L) and nitrofurantoin (I, 64 mg/L) but was susceptible-dose dependent to cefepime (SDD, 8 mg/L). It was also resistant to ertapenem, erythromycin, kanamycin and tetracycline but susceptible to streptomycin, as determined by the Kirby-Bauer disk diffusion method.

### General genome features of the *Raoultella ornithinolytica* strain WLK218

We obtained the complete genome sequence of the *Raoultella ornithinolytica* strain WLK218 using a combination of the PacBio and Illumina sequencing technologies. The *de novo* assembly generated a circular chromosome and six circular plasmids, pWLK-238550, pWLK-107717, pWLK-101716, pWLK-NDM, pWLK-IncN and pWLK-KPC (Table S2 of the Supplementary Materials). Analysis of the whole genome sequence using BacWGSTdb showed that the WLK218 strain contained eighteen antibiotic resistance genes. Most of these genes were carried by plasmid pWLK-238550 (Table S2). The carbapenem resistance genes *bla*_NDM-1_ and *bla*_KPC-2_ were found to be present on two different plasmids, with *bla*_NDM-1_ located on the pWLK-NDM plasmid and *bla*_KPC-2_ located on the pWLK-KPC plasmid.

### Sequence analysis of pWLK-NDM and pWLK-KPC

**Figure 1:**
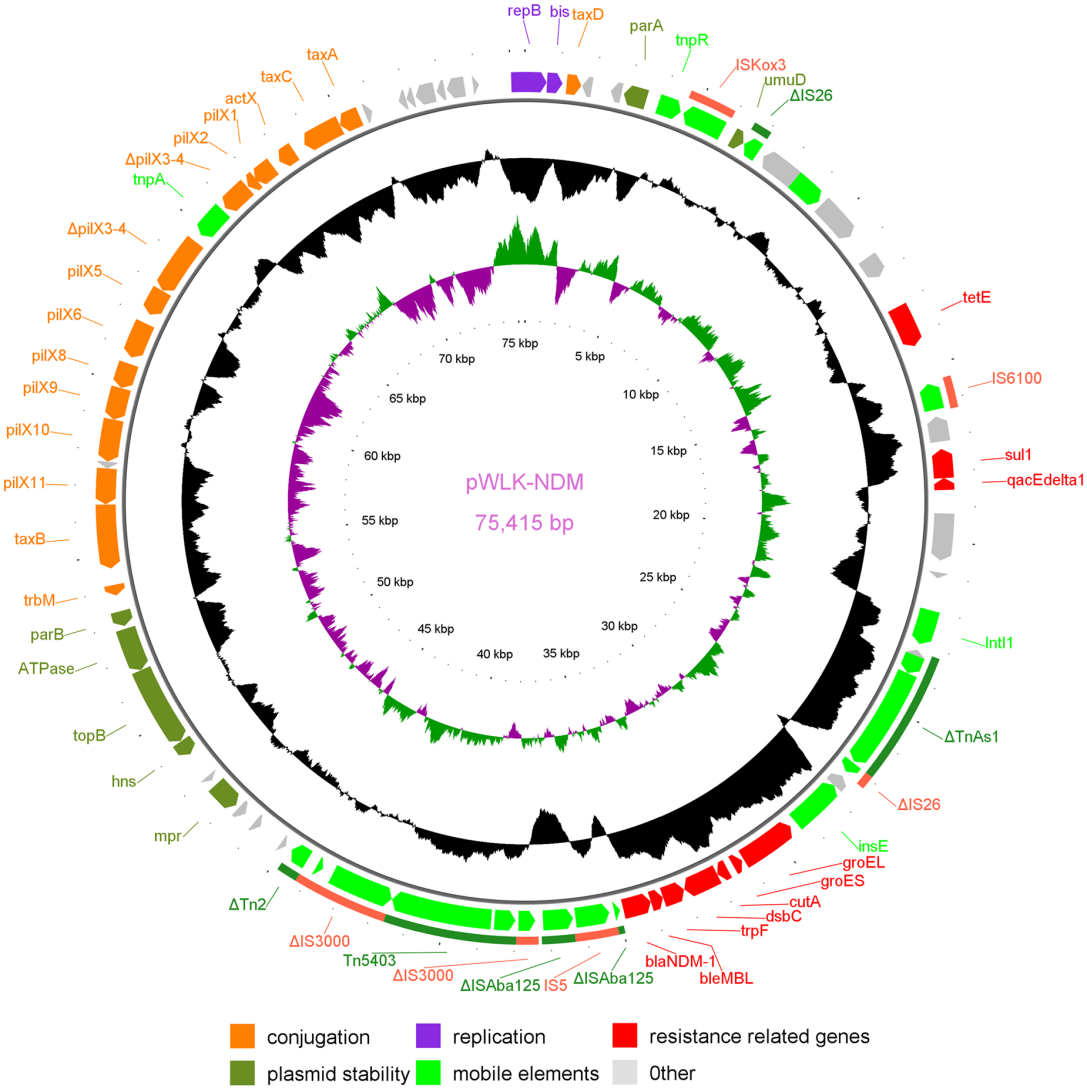
Circular representation of the pWLK-NDM plasmid in the *Raoultella ornithinolytica* strain WLK218. The outer ring displays the positions of the predicted coding sequences, with arrowheads depicting the direction of transcription. The predicted coding sequences are colour-coded depending on their functions. Transposons and insertion sequences are indicated by arcs outside the outer ring. The two inner rings represent the GC content and the GC skew, respectively.

**Figure 2:**
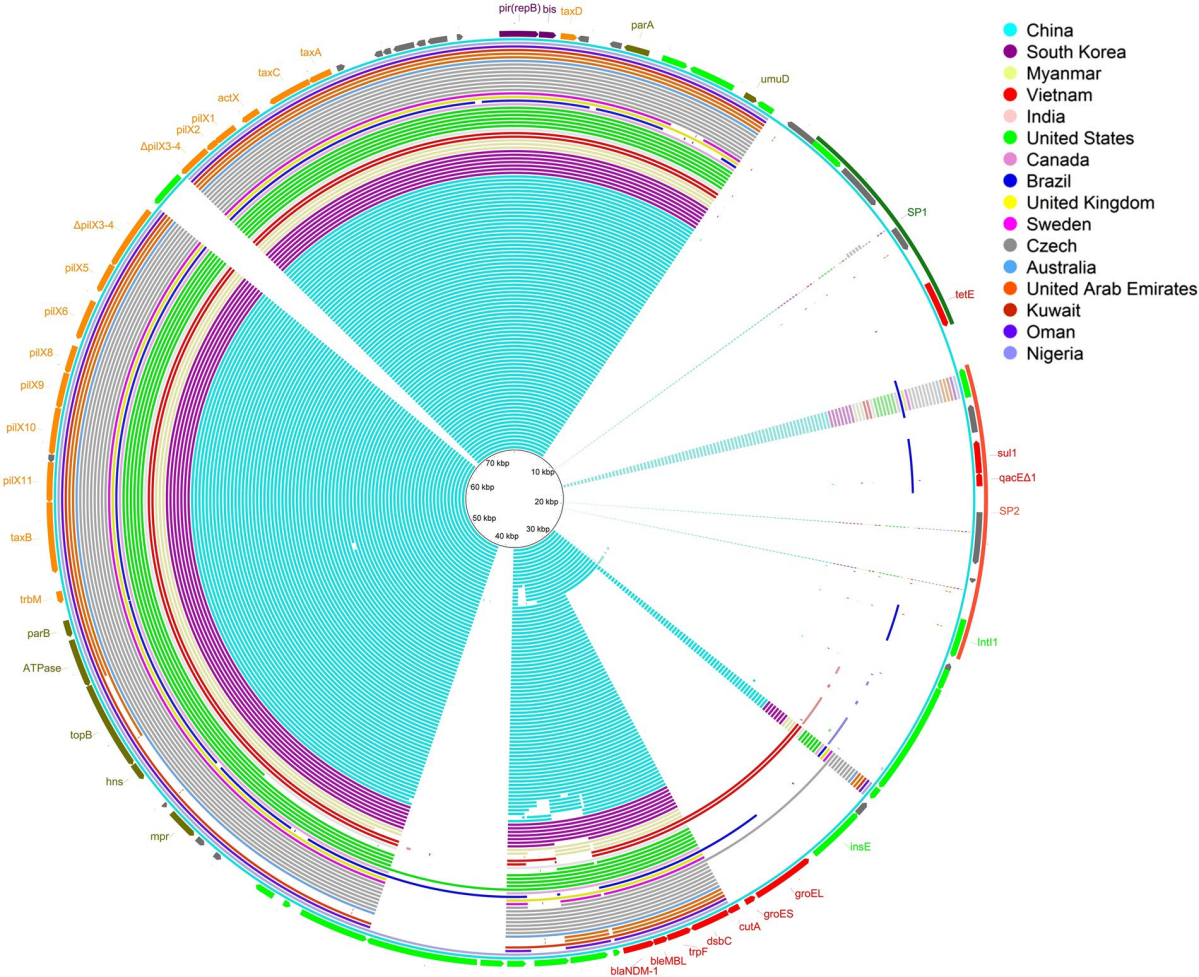
Alignment of the pWLK-NDM plasmid identified in this study with 113 other *bla*_NDM_-carrying IncX3 plasmids isolated from across the world, which include almost all of the *bla*_NDM_-carrying IncX3 plasmids with known sources deposited in the GenBank database. The plasmids are arranged in the order (from inner to outer rings) as described in Table S3 of the Supplementary Materials. The outer ring represents pWLK-NDM. Highlighted on the outer ring are annotations of pWLK-NDM. The map was constructed using BRIG software^36^.

The *bla*_NDM-1_-carrying plasmid pWLK-NDM is 75,415 bp in length, with an average GC content of 50.43%. The IncX3 replicon (100% identity) was identified in pWLK-NDM by using the PlasmidFinder tool. The backbone structure of the pWLK-NDM plasmid is almost identical to the typical IncX3 plasmid structure, which includes the genes encoding proteins involved in replication (*pir* and *bis*), partitioning (*par*A and *par*B), maintenance (*top*B and *hns*), and the conjugative transfer/type IV secretion system (*pil*X, *tax*A, *tax*B, *tax*C and *tax*D), except that the *pil*X3-4 gene of pWLK-NDM is disrupted by a transposase gene with a sequence similar to the insertion sequence IS*Aeca1* (77% identity), which was first described in *Aeromonas caviae* (Figs. 1 and 2). The *bla*_NDM-1_ gene of pWLK-NDM is carried by the genetic structure ΔIS*Aba125*-IS*5*-ΔIS*Aba125*-*bla*_NDM-1_-*ble*_MBL_-*trp*F-*cut*A-*gro*ES-*gro*EL (Figs. 1 and 2), which is nearly identical to the first fully sequenced *bla*_NDM_-harbouring IncX3 plasmid pNDM-HN380^20^ and several other *bla*_NDM-1_-harbouring IncX3 plasmids found in *Enterobacteriaceae* in China (99.99% nucleotide identity and 100% coverage), such as p128379-NDM (GenBank: MF344560), pP10159-1 (GenBank: MF072961), and pNDM-HF727 (GenBank: KF976405).

**Figure 3:**
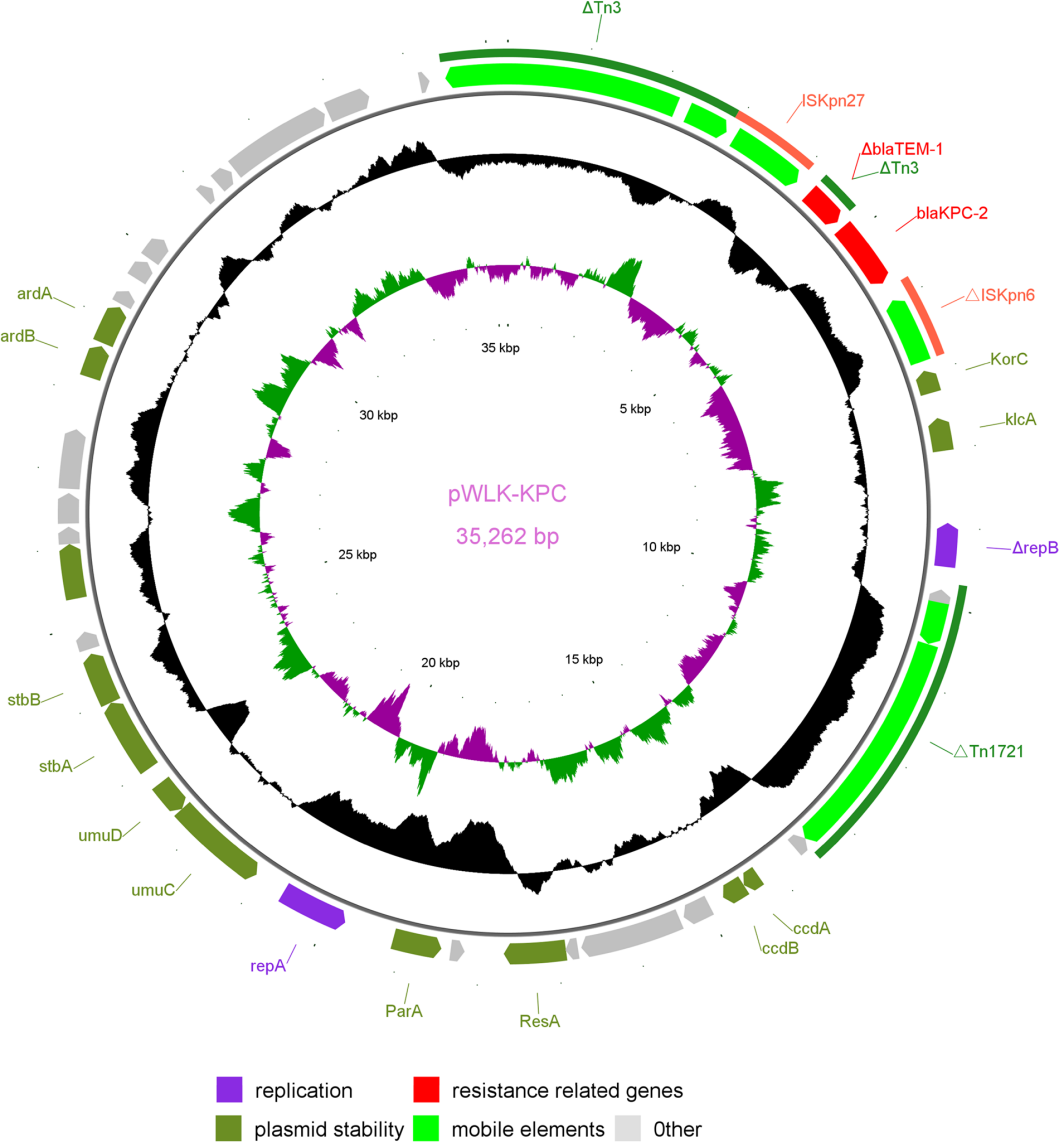
Circular representation of the pWLK-KPC plasmid in the *Raoultella ornithinolytica* strain WLK218. The legend of Fig. 3 is the same as that of Fig. 1.

**Figure 4:**
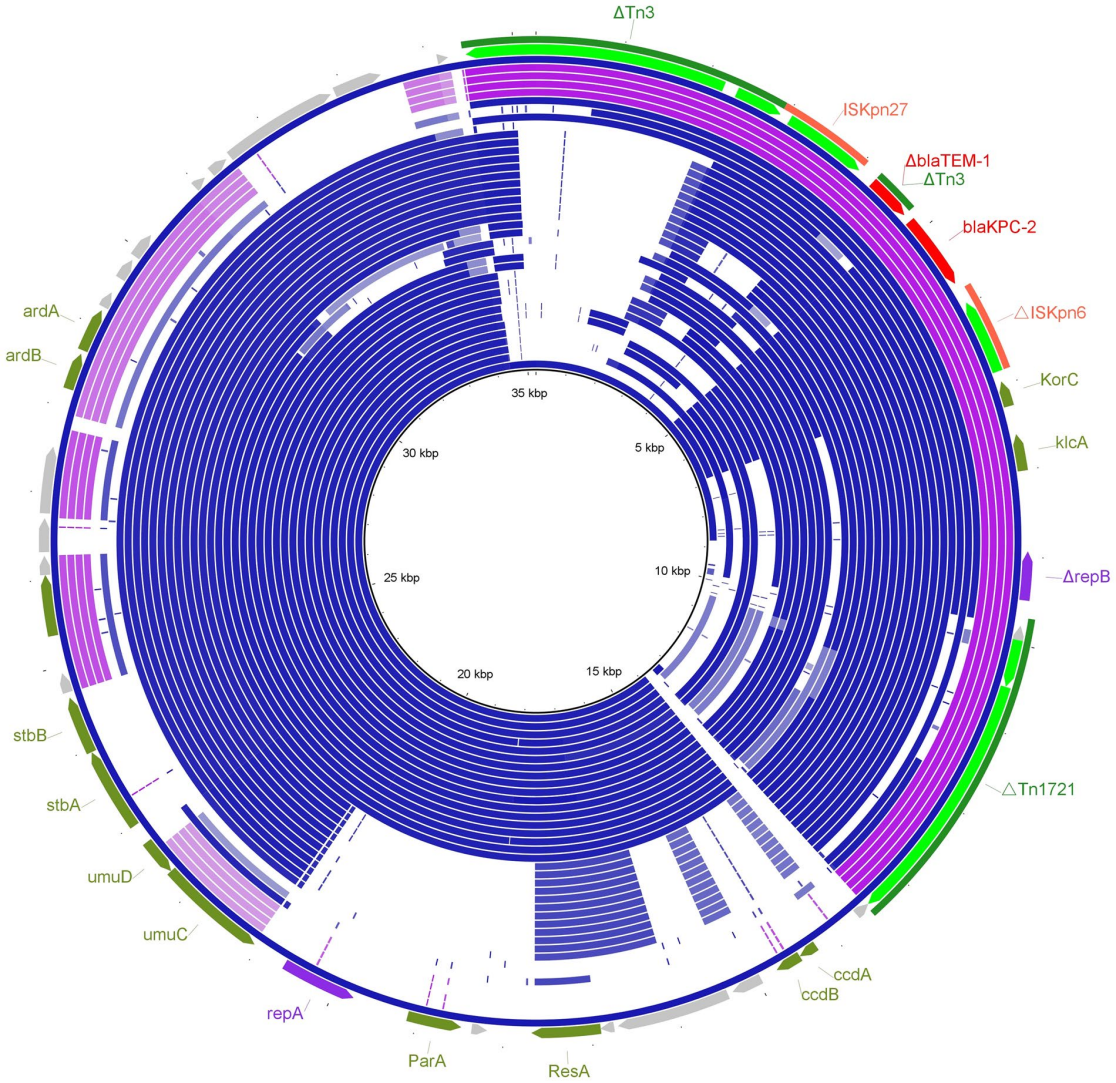
Alignment of the pWLK-KPC plasmid identified in this study with the top 37 other *bla*_KPC_-carrying plasmids from the BLASTN results. The plasmids are arranged in the order (from inner to outer rings) as described in Table S4 of the Supplementary Materials. The outer ring represents pWLK-KPC. Highlighted on the outer ring are annotations of pWLK-KPC. The map was constructed using BRIG software.

The *bla*_KPC-2_-carrying plasmid pWLK-KPC is 35,262 bp in length, with an average GC content of 55.17%. Unlike the pWLK-NDM plasmid, the replicon type of pWLK-KPC plasmid could not be identified by the PlasmidFinder tool, meaning that this plasmid could not be assigned to any known incompatibility group. This suggests that the pWLK-KPC plasmid is a novel type of plasmid. Through sequence analysis, the backbone region of pWLK-KPC was found to contain genes involved in replication and plasmid stability but to lack genes involved in conjugation. The genetic context of *bla*_KPC-2_ on the pWLK-KPC plasmid is comprised of Tn*3*-*tnp*A, Tn*3*-*tnp*R, IS*Kpn27*, Tn*3*-Δ*bla*_TEM-1_*, bla*_KPC-2_, ΔIS*Kpn6*, *kor*C, *klc*A, Δ*rep*B and ΔTn*1721* (Figs. 3 and 4), which is almost identical to that found in four plasmids, pKPC2_EC14653 (GenBank: KP868646), pKPC2_020019 (GenBank: CP028554), pKPC2_EClY2402 (GenBank: KY399972) and pKPC2_EClY2403 (GenBank: KY399973), which were recently identified in human isolates from China.

## Discussion

The *Raoultella* species is closely related to *Klebsiella* spp. This species was originally categorized within the genus *Klebsiella*, but later it was reclassified as *Raoultella* based on comparative analysis of the 16 S rRNA and *rpoB* genes^21^. The *Raoultella* species is mainly found in aquatic environments, but it can also be found in hospital environments and cause severe infections^22^. So far, there have been only three reports of the *bla*_NDM-1_ gene being carried by *Raoultella ornithinolytica*, one involving an isolate from India^22^ and the other two involving isolates from China^23,24^; all three isolates were found in clinical settings and shown to harbour no other carbapenem resistance genes besides *bla*_NDM-1_. There have also been reports describing carbapenem resistance caused by the *bla*_KPC-2_ gene in *Raoultella ornithinolytica* in previous studies^25,26^. However, the coexistence of *bla*_NDM-1_ and *bla*_KPC-2_ has not yet been found in *Raoultella ornithinolytica*. This study reports the coexistence of the *bla*_NDM-1_ and *bla*_KPC-2_ genes in *Raoultella ornithinolytica* for the first time.

In this study, the *Raoultella ornithinolytica* strain WLK218 was found to be resistant to numerous antibiotics and to carry multiple ARGs. Interestingly, most ARGs were carried by plasmids, especially by the pWLK-238550 plasmid. Only two ARGs (*bla*_PLA-1a_ and *fos*A) were carried by the chromosome. Thus, it was the plasmids that mainly contributed to the multidrug resistant phenotypes of the WLK218 strain. In addition, it should be noted that this strain carries a copy of the *aad*A5 gene, which is believed to confer resistance to streptomycin and spectinomycin. However, the WLK218 strain was found to be sensitive to streptomycin. The reasons for this phenomenon are unknown and need further study. Based on sequence analysis, pWLK-NDM and pWLK-KPC were all found to be non-conjugative plasmids. The *bla*_NDM-1_-carrying plasmid pWLK-NDM contains all the genes comprising the IncX3 conjugative apparatus, but the *pil*X3-4 gene is disrupted by a transposase gene, which has rendered this plasmid non-conjugative. The *bla*_KPC-2_-carrying plasmid pWLK-KPC is also a non-conjugative plasmid because this plasmid lacks the genes needed for conjugation.

The *bla*_NDM_ genes have so far been found to be present in multiple species^27^. Plasmids with a variety of replicon types, including IncA/C, IncF, IncH, IncN, IncL/M, IncP, IncR, IncX, and IncY, as well as untypeable plasmids have been identified to be the carriers of the *bla*_NDM_ genes^27^. In China, the *bla*_NDM-1_ gene was first identified in *Acinetobacter baumannii* isolates and found to be carried mainly by *Acinetobacter spp*. initially, while in other countries, *bla*_NDM-1_ was mostly carried by *Enterobacteriaceae*^28^. However, in recent years, reports of NDM-producing *Enterobacteriaceae* isolates in China have been gradually increasing, which mostly resulted from *bla*_NDM_–carrying IncX3 plasmids^29^. In this study, a BLASTN search of the pWLK-NDM sequence against the GenBank database was conducted. A total of 113 *bla*_NDM_-carrying IncX3 plasmids, which include almost all the *bla*_NDM_-carrying IncX3 plasmids with known sources deposited in the GenBank database, were selected and compared with pWLK-NDM using the BLAST Ring Image Generator (BRIG) (Fig. 2). Among the 113 plasmids, most plasmids were isolated from clinical strains and have lengths ranging from 40 to 60 kb. There were only one plasmid isolated from sewage (ring 75), four plasmids isolated from food products (rings 21, 23, 24 and 62) and a few plasmids isolated from animals including pigs, chickens and dogs. Detailed information about the 113 *bla*_NDM_-carrying IncX3 plasmids is summarized in Table S3 of the Supplementary Materials. Compared to the 113 plasmids, pWLK-NDM is much larger in length. As shown in Fig. 2, a 20-kb DNA segment was found to be unique to pWLK-NDM. Through sequence analysis, this 20-kb segment can be divided into two subregions (SP1 and SP2) that share strong homology with different plasmids. SP1 (positions 8360 bp-14297 bp) showed 100% nucleotide identity and 100% query coverage to *Aeromonas hydrophila* subsp. *hydrophila* strain WCHAH045096 plasmid pMCR5_045096 (accession: CP028567), which was isolated from sewage in China. SP2 (positions 15396 bp-23018 bp) showed 99.89% nucleotide identity and 83% query coverage to *Enterobacter cloacae* strain RJ702 plasmid pIMP26 (accession: MH399264), which was isolated from clinical settings in China. Therefore, the formation of pWLK-NDM may involve genetic exchange among different bacterial species from different environments. In addition, the comparison results by BRIG also showed that *bla*_NDM_-carrying IncX3-type plasmids had been spread across the globe, particularly in China, where they were most prevalent. It has been proposed that IncX3-type plasmids may be major vehicles that have mediated the spread of *bla*_NDM_ genes in China. Our finding of pWLK-NDM in river ecosystems is additional evidence of this.

In contrast to the *bla*_NDM_ genes, *bla*_KPC-2_-carrying plasmids are more diverse in terms of structure in China^30^. In this study, the *bla*_KPC-2_-carrying plasmid pWLK-KPC was found to be a novel type of plasmid, which contributes to an improved understanding of the plasmids involved in the dissemination of *bla*_KPC_ genes. The pWLK-KPC sequence was also queried against the GenBank database by BLASTN search. The top 37 *bla*_KPC_ harbouring plasmids with known sources from the BLASTN results were selected and compared with pWLK-KPC using BRIG (Fig. 4). Most of these 37 plasmids are large plasmids and have lengths above 100 kb. Besides, these 37 plasmids were all isolated from clinical strains with two exceptions (rings 7 and 8), which were isolated from faecal samples of wild corvid birds. Detailed information about the 37 *bla*_KPC_-carrying plasmids is summarized in Table S4 of the Supplementary Materials. Compared to the 37 plasmids, pWLK-KPC is much smaller in length. As shown in Fig. 4, the backbone region of pWLK-KPC (positions 14155 bp-34254 bp) was almost identical to the DNA segments from 12 *bla*_KPC_-carrying plasmids (ring 1 to 12). Through sequence analysis, the backbone region of pWLK-KPC was found to be only a small part of the backbone regions of these 12 *bla*_KPC_-carrying plasmids. It is likely that pWLK-KPC is a trimmed-down version of a larger plasmid by homologous recombination, as exemplified by the findings by Conlan *et al*. that pKPN-ff is a trimmed-down version of pKPN-498^31,32^. In addition, the comparison results by BRIG also showed that the *bla*_KPC-2_-containing genetic structure in pWLK-KPC had been mobilized and inserted into different plasmids (purple rings, ring 34 to 37), indicating the high transmission capability of this region.

In this study, six plasmids were observed to coexist in one isolate by whole genome sequencing. The coexistence of such a large number of plasmids reflects that active horizontal genetic transfer events may have taken place before. We propose that pWLK-NDM and pWLK-KPC lost their conjugative functions and became non-conjugative after being transferred into the WLK218 strain. In addition, the coexistence of the *bla*_NDM-1_-carrying plasmid and the *bla*_KPC-2_-carrying plasmid in the same isolate is worrying, as this may generate a new mobile platform that carries both genes, which will facilitate the spread of carbapenem resistance genes. This is exemplified by the recent finding by Sun *et al*. that the *mcr-1* and *bla*_NDM-5_ genes are co-located on the same plasmid^33^. More worrisome is the fact that aquatic sediments represent an important environmental matrix within which genetic transfer and recombination occur^34,35^, raising the possibility that the *Raoultella ornithinolytica* strain WLKW218 will acquire additional ARGs and become pan-drug resistant.

### Conclusions

We report the isolation and characterization of a *Raoultella ornithinolytica* strain harbouring both *bla*_NDM-1_ and *bla*_KPC-2_ from urban river sediment. The characterization of this strain will not only provide new insights into the genetic platforms contributing to the dissemination of the *bla*_NDM-1_ and *bla*_KPC-2_ genes but also expand our knowledge of the environmental dissemination of these genes. This study highlights the potential dissemination of *bla*_NDM-1_ and *bla*_KPC-2_ genes throughout the environment, which should be of great concern.

## Supplementary information

Supplementary Information.
