# Supplementary material for: Coexistence of the blaNDM-1-carrying plasmid pWLK-NDM and the blaKPC-2-carrying plasmid pWLK-KPC in a Raoultella ornithinolytica isolate
Source: Sci Rep. 2020 Feb 11;10:2360. doi: 10.1038/s41598-020-59341-4 (PMC7012882; doi:10.1038/s41598-020-59341-4)
Supplement: Supplementary file 1 — Supplementary Information. [file 41598_2020_59341_MOESM1_ESM.pdf]

Supplementary Materials for

Coexistence of the *bla*$_{NDM-1}$-carrying plasmid pWLK-NDM and the *bla*$_{KPC-2}$-carrying plasmid pWLK-KPC in a *Raoultella ornithinolytica* isolate

Bingjun Dang[1, *], Haoyang Zhang[1], Ziwei Li[1], Shuanglong Ma[2] & Zicheng Xu[1, *]

[1]College of Tobacco Science, Henan Agricultural University, Zhengzhou 450002, China.

[2]College of Resources and Environmental Sciences, Henan Agricultural University, Zhengzhou 450002, China.

Corresponding Authors

*Phone: +86 (0371) 63555763. E-mail: dangbingjun@henau.edu.cn

*Phone: +86 (0371) 63555916. E-mail: zichengxu@henau.edu.cn

Table S1 Primers used for the screening of carbapenem resistance genes and identification of bacterial isolates.

Primers	Primer sequence(5' to 3')	Target genes	Amplicon size (bp)	Reference
KPC-F	TGTCACTGTATCGCCGTCTAG	*bla*$_{\text{KPC-2}}$	881	1
KPC-R	TTACTGCCCGTTGACGCCCAATCC			
NDM-F	GGTTTGGCGATCTGGTTTTC	*bla*$_{\text{NDM-1}}$	621	2
NDM-R	CGGAATGGCTCATCACGATC			
IMP-F	GTTTATGTTCATACWTCG	*bla*$_{\text{IMP}}$	432	3
IMP-R	GGTTTAAYAAAACAACCAC			
27-F	AGAGTTTGATCCTGGCTCAG	16S rRNA	1465	4
1492-R	GGTTACCTTGTTACGACTT			

Table S2. Detailed information on the genome of the *Raoultella ornithinolytica* strain WLK218.

Name	Sequence type	Sequence topology	Length (bp)	GC content (%)	ARGs and identity
chromosome	chromosome	circular	5,623,484	55.43	*bla*$_{\text{PLA-1a}}$ (94.18%), *fos*A (99.52%)
pWLK-238550	plasmid	circular	238,550	46.48	*aad*A5 (100%), *sul*1 (100%, 2 copies), *arm*A (100%, 2 copies), *dfr*A1 (100%), *msr*(E) (100%), *mph*(E) (100%), *arr-3* (100%), *cat*B3 (99.84%), *bla*$_{\text{OXA-1}}$ (100%), *aac(6')-Ib-cr* (100%)
pWLK-107717	plasmid	circular	107,717	51.98	none
pWLK-101716	plasmid	circular	101,716	52.81	none
pWLK-NDM	plasmid	circular	75,415	50.43	*bla*$_{\text{NDM-1}}$ (100%), *sul*1 (100%), *tet*E (99.26%)
pWLK-IncN	plasmid	circular	55,184	50.90	none
pWLK-KPC	plasmid	circular	35,262	55.17	*bla*$_{\text{KPC-2}}$ (100%)

Table S3 Detailed information of the 113 *bla*$_{NDM}$-carrying IncX3 plasmids.

order	accession	country	size	source
1	KP987216	China	53,489 bp	clinical
2	MF344560	China	54,186 bp	clinical
3	KX470734	China	54,035 bp	clinical
4	KF976405	China	54,035 bp	clinical
5	JX104760	China	54,035 bp	clinical
6	MF072961	China	54,034 bp	clinical
7	MF344558	China	56,044 bp	clinical
8	KY913899	China	55,246 bp	clinical
9	CP028786	China	54,035 bp	clinical
10	KX094555	China	54,035 bp	clinical
11	KY978629	China	53,793 bp	clinical
12	MH234505	China	53,097 bp	clinical
13	CP034323	China	53,144 bp	clinical
14	KF877335	China	53,134 bp	clinical
15	CP029386	China	52,989 bp	clinical
16	MF415608	China	60,125 bp	clinical
17	MH523639	China	46,161 bp	clinical
18	CP032424	China	46,161 bp	clinical
19	CP032889	China	46,161 bp	clinical
20	CP034965	China	46,161 bp	clinical
21	MG825384	China	46,161 bp	food, chicken
22	CP019073	China	46,145 bp	clinical
23	MG825368	China	46,161 bp	food, pork
24	MG825382	China	46,161 bp	food, pork
25	KX833071	China	46,161 bp	anal swab, chicken
26	MF679143	China	46,161 bp	clinical
27	KT824791	China	46,164 bp	clinical
28	CP035125	China	46,161 bp	clinical
29	MG545911	China	46,145 bp	clinical
30	KX507346	China	46,165 bp	pig faeces
31	CP022351	China	46,149 bp	clinical
32	CP023188	China	46,146 bp	clinical
33	CP034591	China	45,650 bp	anal swab
34	CP034737	China	46,259 bp	anal swab
35	MG773377	China	46,192 bp	clinical
36	MF458176	China	46,161 bp	anal swab, pig
37	MH234509	China	47,474 bp	clinical

38	MH234508	China	46,161 bp	clinical
39	MH234507	China	46,161 bp	clinical
40	MH234506	China	46,161 bp	clinical
41	MH234504	China	46,161 bp	clinical
42	MH234503	China	46,161 bp	clinical
43	MH234501	China	46,161 bp	clinical
44	MH234499	China	47,849 bp	clinical
45	MH234498	China	46,161 bp	clinical
46	MH234497	China	46,161 bp	clinical
47	KU167608	China	46,161 bp	clinical
48	KU167609	China	46,161 bp	clinical
49	CP025948	China	46,161 bp	clinical
50	CP033057	China	46,161 bp	clinical
51	CP026577	China	46,161 bp	clinical
52	CP028577	China	46,161 bp	clinical
53	CP034957	China	46,161 bp	clinical
54	CP033399	China	46,161 bp	clinical
55	CP031725	China	46,161 bp	clinical
56	CP036179	China	46,161 bp	clinical
57	KU761328	China	46,161 bp	clinical
58	KY435936	China	47,337 bp	clinical
59	MH161191	China	46,161 bp	rectal swab, chicken
60	CP028718	China	46,161 bp	clinical
61	CP029245	China	46,161 bp	chicken faeces
62	CP028705	China	46,161 bp	food, cucumber
63	CP014006	China	46,161 bp	clinical
64	MF547510	China	46,161 bp	rectal swab, pig
65	MF547509	China	46,161 bp	rectal swab, pig
66	MF547507	China	46,161 bp	rectal swab, pig
67	MF547508	China	46,161 bp	rectal swab, pig
68	MH234500	China	46,161 bp	clinical
69	CP036312	China	46,161 bp	clinical
70	CP036205	China	51,321 bp	clinical
71	MF547511	China	45,546 bp	rectal swab, pig
72	MH234502	China	45,547 bp	clinical
73	CP025215	China	68,637 bp	clinical
74	CP028536	China	45,048 bp	clinical
75	CP027204	China	44,088 bp	sewage
76	MH061381	China	45,739 bp	clinical

77	CP024820	South Korea	46,161 bp	clinical
78	CP024833	South Korea	46,137 bp	clinical
79	CP024828	South Korea	51,455 bp	clinical
80	CP024825	South Korea	46,161 bp	clinical
81	CP024818	South Korea	46,161 bp	clinical
82	MH094148	South Korea	46,163 bp	rectal swab, dog
83	CP024814	South Korea	44,962 bp	clinical
84	AP018146	Myanmar	46,161 bp	clinical
85	AP018141	Myanmar	45,122 bp	clinical
86	AP018142	Myanmar	43,534 bp	clinical
87	AP018571	Viet Nam	52,715 bp	clinical
88	CP020090	Viet Nam	62,470 bp	clinical
89	KF220657	India	46,253 bp	clinical
90	KX447767	USA	39,520 bp	clinical
91	CP021715	USA	43,378 bp	clinical
92	CP021534	USA	46,161 bp	clinical
93	CP021738	USA	46,159 bp	clinical
94	CP021692	USA	46,161 bp	clinical
95	CP021682	USA	49,828 bp	clinical
96	CP012990	Canada	46,161 bp	clinical
97	KR822247	Brazil	74,852 bp	clinical
98	CP031138	United Kingdom	46,157 bp	clinical
99	CP023260	Sweden	44,106 bp	clinical
100	MG252893	Czech	53,051 bp	clinical
101	MG833403	Czech	53,683 bp	clinical
102	MG833402	Czech	53,683 bp	clinical
103	MG252892	Czech	53,683 bp	clinical
104	MG833404	Czech	53,683 bp	clinical
105	MG833405	Czech	53,683 bp	clinical
106	MG252891	Czech	46,161 bp	clinical
107	MG833406	Czech	46,161 bp	clinical
108	KM400601	Australia	46,253 bp	clinical
109	KX214671	United Arab Emirates	37,070 bp	clinical
110	KX214670	United Arab Emirates	34,403 bp	clinical
111	KX214669	Kuwait	46,161 bp	clinical
112	KP776609	Oman	45,122 bp	clinical
113	CP034133	Nigeria	52,372 bp	clinical

Table S4 Detailed information of the 37 *bla*$_{KPC}$-carrying plasmids.

order	accession	country	size	source
1	CP028389	China	166034 bp	clinical
2	CP014765	USA	106559 bp	clinical
3	CP018999	China	146162 bp	clinical
4	CP034777	Canada	109503 bp	clinical
5	KP987218	China	105008 bp	clinical
6	KX236178	China	121348 bp	clinical
7	KY093013	Canada	134521 bp	faecal samples of wild corvid birds
8	KY093014	Canada	108772 bp	faecal samples of wild corvid birds
9	KY270849	China	143538 bp	clinical
10	MG764550	China	164198 bp	clinical
11	MH477636	China	163588 bp	clinical
12	MH917122	China	106201 bp	clinical
13	CP036306	China	149519 bp	clinical
14	CP038003	China	149518 bp	clinical
15	KU295132	USA	101915 bp	clinical
16	CP008791	USA	205586 bp	clinical
17	MF918372	China	117697 bp	clinical
18	MG764553	China	153274 bp	clinical
19	CP018455	China	162552 bp	clinical
20	CP026131	China	164510 bp	clinical
21	CP026141	China	164501 bp	clinical
22	CP029381	China	146790 bp	clinical
23	CP033956	China	133772 bp	clinical
24	CP034125	China	109179 bp	clinical
25	CP034417	China	128299 bp	clinical
26	CP041375	China	134972 bp	clinical
27	KT185451	China	151466 bp	clinical
28	MF168402	China	146103 bp	clinical
29	MF168404	China	151653 bp	clinical
30	KY689238	China	85874 bp	clinical
31	MF156712	China	46123 bp	clinical
32	MH192342	China	95734 bp	clinical
33	MN268581	China	60195 bp	clinical
34	KY399973	China	88213 bp	clinical
35	CP028554	China	89738 bp	clinical
36	KP868646	China	88214 bp	clinical
37	KY399972	China	88213 bp	clinical

References

1 Kaczmarek, F. M., Dib-Hajj, F., Shang, W. & Gootz, T. D. High-level carbapenem resistance in a *Klebsiella pneumoniae* clinical isolate is due to the combination of *bla*$_{\text{ACT-1}}$ β-lactamase production, porin OmpK35/36 insertional inactivation, and down-regulation of the phosphate transport porin PhoE. *Antimicrob. Agents Chemother.* **50,** 3396-3406 (2006).

2 Poirel, L., Walsh, T. R., Cuvillier, V. & Nordmann, P. Multiplex PCR for detection of acquired carbapenemase genes. *Diagn. Microbiol. Infect. Dis.* **70,** 119-123 (2011).

3 Hujer, K. M. *et al.* Analysis of antibiotic resistance genes in multidrug-resistant *Acinetobacter* sp. isolates from military and civilian patients treated at the Walter Reed Army Medical Center. *Antimicrob. Agents Chemother.* **50,** 4114-4123 (2006).

4 Crisafi, F. *et al.* Comparison of *16SrDNA* and *toxR* genes as targets for detection of *Vibrio anguillarum* in *Dicentrarchus labrax* kidney and liver. *Res. Microbiol.* **162,** 223-230 (2011).
